# Sequence determinants of RNA G‐quadruplex unfolding by Arg‐rich regions

**DOI:** 10.1002/1873-3468.70274

**Published:** 2026-01-09

**Authors:** Naiduwadura Ivon Upekala De Silva, Puspa Kunwar, Md Ibnul Rifat Rahman, Joanna Koryo Kwao, Nathan Lehman, Zihan Zhang, Trenton Paul, Claire Cheng, Nicholas Truex, Hui‐Ting Lee, Jun Zhang

**Affiliations:** ^1^ Department of Chemistry University of Alabama at Birmingham AL USA; ^2^ Department of Chemistry and Biochemistry University of South Carolina Columbia SC USA; ^3^ Livingston High School NJ USA

**Keywords:** Arg‐rich peptides, G‐quadruplex, splicing, SR proteins, SR‐related proteins, unfolding

## Abstract

RNA sequences with the potential to form G‐quadruplexes (rGQs) are widespread but largely unfolded in cells by unknown mechanisms. rGQ folding status is a critical regulator of RNA splicing and translation. We show that rGQs can be unfolded by SR proteins, SR‐related proteins, and other Arg‐rich proteins, including SRSF1, SRSF3, SRSF9, U1‐70K, and U2AF1. The length and composition of Arg‐rich regions are key determinants of this activity: Arg residues are the primary drivers, while acidic residues attenuate the unfolding activity. To unfold ARPC2 rGQ, at least 13 Arg residues are required. Our findings identify Arg‐rich proteins as previously unrecognized, helicase‐independent regulators of rGQ structures, with potential broad impacts on RNA processing that merit further investigation.

## Graphical abstract

We show that Arg‐rich peptides selectively unfold RNA G‐quadruplexes, but not RNA stem‐loops or DNA/RNA duplexes. This length‐dependent activity is inhibited by acidic residues and is conserved among SR and SR‐related proteins (SRSF1, SRSF3, SRSF9, U1‐70K, and U2AF1).

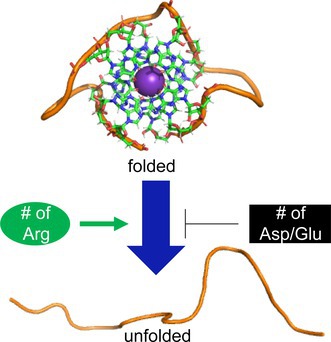

## Abbreviations

**AFPS**, automated flow peptide synthesis

**FP**, fluorescence polarization

***K***_**D**_, dissociation constant

**MD**, molecular dynamics

**rGQs**, RNA G‐quadruplexes

**RS**, Arg/Ser‐rich region

**smFRET**, single‐molecule Förster resonance energy transfer

**SR**, Ser/Arg‐rich splicing factor

**Fig. 1:**
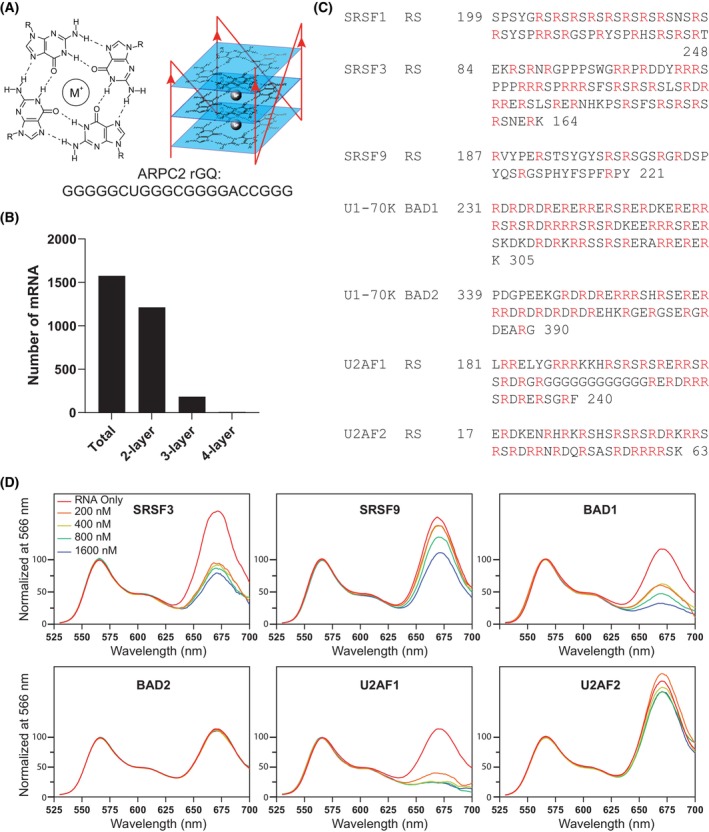
SR and SR‐related proteins are capable of unfolding the ARPC2 rGQ. (A) Schematic representation of an RNA G‐quadruplex (rGQ). M^+^ denotes monovalent cations positioned between G‐tetrad layers, coordinating with carbonyl oxygen atoms. A parallel GQ structure is shown on the right. The sequence of the ARPC2 rGQ is shown below. (B) Distribution of 2‐layer, 3‐layer, and 4‐layer rGQs among mRNAs regulated by SRSF1. (C) Amino acid sequences of RS regions with Arg highlighted by red from SRSF1, SRSF3, SRSF9, U1‐70K (BAD1 and BAD2), U2AF1, and U2AF2. (D) Fluorescence spectra of Cy5/Cy3‐labeled ARPC2 rGQ in 25 mm KCl normalized to the Cy3 emission maximum at 566 nm. All spectra were recorded using excitation at 500 nm. ARPC2 rGQ was used at 200 nm, and the concentrations of SR or SR‐related proteins are indicated. The spectra were averaged from five scans.

G‐quadruplexes (GQ) are ubiquitous in the human genome. In a GQ, four guanine bases are arranged through Hoogsteen base pairing to form a plane known as a G‐tetrad that stacks in a multilayered fashion (Fig. 1A). Three or more layers of G‐tetrads stack to create a GQ structure, which is further stabilized by monovalent cations that bind to carbonyl oxygen atoms of guanines in the order of K^+^ > Na^+^ > Li^+^. This structure requires GQ‐forming sequences to share a formula of G_x_N_1–7_G_x_N_1–7_G_x_N_1–7_G_x_, in which x ≥ 3 and N represents any nucleotide. According to this formula, recent studies have identified around 300 000–700 000 GQ‐forming motifs in the human genome [1, 2]. GQs demonstrate considerable diversity. A GQ can form with only two layers of G‐tetrads [3, 4]. Imperfect GQs can also form with a G missing in one G‐tetrad or a bulge incorporated between G‐tetrad layers [5]. GQs with two‐layer G‐tetrads or imperfections usually have a much lower stability [3, 4, 5, 6].

GQ structures can arise from both DNA and RNA sequences. rGQs influence RNA transcription, mRNA stability, alternative splicing mechanisms, and ribosomal translation [7]. rGQs are enriched near splice junctions [8]. Intronic rGQs are correlated with the skipping of downstream exons in human beings [9]. However, case studies on SLC6A17, NAV2, and CD44 have found that rGQs in introns promote exon inclusion [8, 10]. The regulatory role of rGQs on splicing is likely associated with various RNA‐binding proteins [8, 10]. Besides their roles in regulating RNA splicing, rGQs usually inhibit ribosomal translation [11, 12]. GQ‐promoting sequences are prevalent in RNA transcripts, but only a small portion of them yield functional GQ structures within cells [13]. Regulation of GQ formation is especially important for GQ structures with exceptional stability. Various helicases such as DDX5 and DHX36 can unwind GQs in an ATP‐dependent way [14, 15]. In addition, several RNA‐binding proteins participate in this regulation. For example, hnRNPH1 inhibits GQ formation via its RNA‐recognition motifs [16]. However, due to the specificity of hnRNPH1, this only prevents GQ formation for a small subset of RNA. Dysregulation of the rGQ level is associated with diseases. Recent studies have observed the accumulation of rGQs in aging and Alzheimer's disease and the association of rGQs with α‐synuclein fibrillation [17, 18]. Considering the prevalence of RNA sequences with potential to form rGQs, it is of general interest to understand how cells globally unfold rGQs in basic research, and to find tools to prevent rGQ formation for therapeutic purposes.

We have recently found that SRSF1 unfolds rGQs through its Arg/Ser‐rich (RS) region and that the RS region exhibits RNA‐binding specificity by preferentially recognizing guanine‐ or purine‐rich sequences [12]. This observation is further supported by a similar finding for the Arg‐rich region of TRA2 [19]. SRSF1 is a member of the SR protein family that consists of 12 members, each containing RS regions. RS regions are also shared by SR‐related proteins, a broader protein family containing over 100 proteins with well‐known members such as U1‐70K, U2AF1, and U2AF2 [20, 21]. These SR or SR‐related proteins are involved in RNA splicing. It is of interest to know whether other SR proteins or SR‐related proteins also unwind rGQs. In addition, our previous study showed that Arg, rather than Ser, in the RS region of SRSF1 is critical for unwinding activity. We hypothesize that the rGQ unfolding activity may be shared by proteins containing Arg‐rich regions, which further expands the spectrum of the proteins with such activity [22]. Therefore, we also wondered whether other Arg‐rich regions with different accompanying amino acids could also unfold rGQs.

In this study, we investigated rGQ unfolding with fragments of SR or SR‐related proteins that contain varying amino acid sequence compositions and lengths. We studied unfolding activity in other SR or SR‐related proteins, including SRSF3, SRSF9, U1‐70K, and U2AF1. We found that important factors for the rGQ‐unfolding activity include the number of Arg residues and the net charges. For the rGQ in the 5′ untranslated region of actin related protein complex subunit 2 (ARPC2 rGQ) as a model, it can be unfolded by a peptide containing more than 13 Arg residues. The polarity of the residues adjacent to Arg residues has a secondary impact, except for acidic amino acids, which neutralize the positive charge of Arg and attenuate the rGQ‐unfolding activity. Using bulk and single‐molecule FRET, we further found that the unfolding activity of the Arg‐rich region is not applicable to stem‐loop or RNA/DNA hybrid RNA structures. Taken together, our research reveals that unfolding activity is shared among many proteins with Arg‐rich regions and is specific to rGQ structures.

## Materials and methods

### Purification of SRSF1 RS domain (residues 199–248), RS1 domain (residues 199–225), U1‐70K BAD1, and U1‐70K BAD2

These constructs were expressed in *E. coli* BL21(DE3) Star cells grown overnight at 16 °C in LB medium. Protein expression was induced with 0.5 mm IPTG when cultures reached an OD_600_ of 0.6 at 37 °C. Cell pellets were resuspended in lysis buffer composed of 20 mm Tris–HCl, pH 7.5, 25 mm imidazole, 0.1 mm TCEP, and 6 m guanidinium hydrochloride. Cell lysis was achieved through three cycles of freezing and thawing, followed by sonication. The cell lysate was centrifuged at 23 710 ***g*** for 40 min using a Beckman Coulter Avanti JXN‐26 centrifuge. The resulting supernatant was applied to a 5‐mL HisPur Ni‐NTA affinity column and washed with 200 mL of wash buffer containing 20 mm Tris–HCl, pH 7.5, 25 mm imidazole, and 0.2 mm TCEP. The protein was eluted from the Ni‐resin using 30 mL of buffer consisting of 500 mm Arg/Glu, pH 7.0, 500 mm imidazole, 1 mm TCEP, and a tablet of protease inhibitor cocktail. The eluted protein was treated with 2 μg·mL^−1^ Ulp1 protease for 1 h at 37 °C to remove the SUMO tag and then diluted twofold with buffer A (20 mm sodium acetate, pH 5.0, 0.2 mm TCEP). The diluted sample was loaded onto a 5‐mL HiTrap SP cation exchange column and eluted using a linear gradient with buffer B containing 20 mm sodium acetate (pH 5.0), 2 m NaCl, and 0.2 mm TCEP. The RS domain typically eluted around 30% buffer B. Eluted fractions containing the target protein were combined, concentrated, and buffer‐exchanged into 20 mm Tris–HCl, pH 7.5 with 0.2 mm TCEP. The RS1 domain of SRSF1 was purified using the same procedure. U1‐70K BAD1 and BAD2 were purified in a similar procedure with minor modifications described in our previous study [23].

### SRSF3 purification

The human SRSF3 (1–164) gene was cloned into the pSMT3 vector, and truncated constructs such as SRSF3 (1–148) and the RS tail (85–164) were generated using mutagenesis PCR. These constructs were expressed in *E. coli* BL21(DE3) Star cells grown overnight at 16 °C in LB medium. Protein expression was induced with 0.5 mm IPTG when cultures reached an OD_600_ of 0.6 at 37 °C. Cells were harvested and resuspended in a lysis buffer containing 20 mm Tris–HCl, pH 7.5, 6 m guanidinium HCl, 25 mm imidazole, 0.2 mm TCEP, 1 mm PMSF, 0.5 mg·mL^−1^ lysozyme, and one tablet of protease inhibitor cocktail, followed by two freeze–thaw cycles. The cells were lysed by sonication and centrifuged at 23710 ***g*** for 40 min at 4 °C to remove debris. The supernatant was applied to 5 mL of Nickel‐NTA resin, washed with high‐salt buffer (20 mm Tris, pH 7.5, 8 m urea, 2 m NaCl, 0.2 mm TCEP) and a second wash buffer (20 mm Tris–HCl, pH 7.5, 8 m urea, 50 mm imidazole, 0.2 mm TCEP), and eluted using 20 mm MES, pH 6.5, 8 m urea, 0.5 m Arg/Glu, 500 mm imidazole, and 0.2 mm TCEP. The eluted protein was further purified using a 5‐mL HiTrap SP column after fourfold dilution with SP buffer A (20 mm CHES, pH 8.6, 4 m urea, 0.2 mm TCEP) and eluted with a gradient of SP buffer B (20 mm CAPS, pH 11.1, 4 m urea, 0.5 m Arg, 2 m NaCl, 0.2 mm TCEP). Final purification was performed using a Superdex 75 Increase 10/300 GL column equilibrated with 400 mm Arg/Glu, pH 7.0 and 0.2 mm TCEP. For the RS tail construct, SP purification was performed after dilution in 20 mm acetate buffer (pH 5.0, 0.2 mm TCEP), followed by elution with a 0–2 m NaCl gradient and buffer exchange into 400 mm Arg/Glu, pH 7.0 with 0.2 mm TCEP.

### Purification and refolding of U2AF1

The DNA sequence encoding human U2AF1 was cloned into the pSMT3 vector downstream of an N‐terminal His_6_‐SUMO tag. The plasmid was transformed into *E. coli* BL21‐CodonPlus (DE3) cells and expressed in LB medium supplemented with 50 μg·mL^−1^ kanamycin and 50 μg·mL^−1^ chloramphenicol. Protein expression was induced with 0.5 mm IPTG at an optical density (OD_600_) of 0.6, followed by overnight incubation at 22 °C. Cells were harvested by centrifugation and resuspended in lysis buffer containing 6 m guanidinium‐HCl, 2 m NaCl, 20 mm Tris–HCl, pH 7.5, 25 mm imidazole, 0.2 mm TCEP, and 0.5 mg·mL^−1^ lysozyme. Lysis was performed by sonication in an ice‐water bath. The lysate was clarified by centrifugation at 23 710 ***g*** for 40 min at 4 °C, and the supernatant was applied to a 5‐mL Ni‐NTA affinity column. On‐column refolding and removal of contaminants were carried out by sequential washes (50 mL each) using refolding buffer containing decreasing concentrations of guanidinium‐HCl (4.8, 3.6, 2.4, 1.2, and 0 m) in 20 mm Tris–HCl, pH 7.5, 2 m NaCl, 25 mm imidazole, 0.2 mm TCEP, and 1 μm ZnCl_2_. The His_6_‐SUMO‐U2AF1 fusion protein was eluted with 30 mL of 20 mm MES, pH 6.0, 0.5 m Arg/Glu, 0.5 m imidazole, and 0.2 mm TCEP. The eluted protein was concentrated and further purified by size‐exclusion chromatography using a Superdex 75 pg 16/600 column (Cytiva) equilibrated with 400 mm Arg/Glu, pH 6.0 and 0.2 mm TCEP. Protein purity was confirmed by SDS/PAGE.

### Purification of U2AF2

Human U2AF2 gene was cloned into pSMT3 vector downstream of an N‐terminal His_6_‐SUMO tag. The plasmid was then transformed into *E. coli* BL21‐CodonPlus(DE3) competent cells and cultured in 4 L of LB medium supplemented with 50 μg·mL^−1^ of kanamycin and 50 μg·mL^−1^ of chloramphenicol at 37 °C. Protein expression was induced with 0.25 mm IPTG at an optic density of 0.6, followed by overnight incubation at 25 °C. Cells were collected and resuspended in lysis/wash buffer: 20 mm Tris–HCl, pH 7.5, 2 m NaCl, 25 mm imidazole, 0.2 mm TCEP, 5% glycerol, then sonicated in an ice‐water bath. The lysate was clarified by centrifugation at 23 710 ***g*** for 40 min at 4 °C. The supernatant was applied to a 5‐mL Ni‐NTA resin column, then washed with 200 mL of lysis/wash buffer. The U2AF2 protein was eluted with 25 mL of elution buffer (20 mm Tris–HCl, pH 7.5, 500 mm Arg/Glu, 250 mm imidazole, 0.2 mm TCEP, 5% glycerol). N‐terminal His_6_‐SUMO tag was cleaved off by 200 μg of house‐made Ulp1 at 37 °C for 30 min. The cleaved sample was diluted with 100 mL of Heparin buffer A (20 mm HEPES, pH 7.0, 0.2 mm TCEP) and loaded onto a 5‐mL Heparin‐HP column. Protein was eluted from the heparin column using gradient elution. Peak fractions were collected and loaded onto a Superdex 75 pg 16/600 column (Cytiva) equilibrated in 400 mm Arg/Glu, pH 7.5 with 0.2 mm TCEP. Peak fractions were concentrated using a 30‐kDa concentrator.

### Synthetic RNA oligonucleotides

Synthetic ARPC2 rGQ, 5‐Cy5‐UGGGGGCUGGGCGGGGACCGGGU‐Cy3‐3′, stem‐loop RNA: 5′‐Cy5‐UCUCUCUCUCGAGAGAGAGA‐Cy3Sp/‐3′ were synthesized by Integrated DNA Technologies and dissolved in H_2_O to a concentration of 100 μm, then diluted 10‐fold in buffer containing 20 mm Tris–HCl, pH 7.5, 25 mm KCl, and 0.1 mm EDTA. The RNA was folded using a thermocycler by heating at 95 °C for 5 min, followed by an annealing gradient to 4 °C at a rate of 1 °C·min^−1^.

### Fluorescence spectroscopy

The Cy3–Cy5 labeled RNA (200 nm) was dissolved in 100 μL of buffer containing 25 mm KCl or LiCl, 0.25 m Arg/Glu, 20 mm Tris–HCl, pH 7.5, 0.1 mm EDTA, and 0.1 mm TCEP. Protein stock solutions (20 μm), dissolved in 20 mm Tris–HCl, pH 7.5, 0.4 m Arg/Glu, and 0.1 mm TCEP, were titrated into ARPC2 rGQ. Fluorescence emission spectra were recorded from 530 to 700 nm using a Cytation 5 in black flat‐bottom 96‐well plates (Costar) at 25 °C. Some samples exhibited phase separation upon titration of GQ RNA. To eliminate interference from phase separation, the protein‐RNA mixtures were centrifuged at 16 000 ***g*** for 10 min, and the supernatants were used for fluorescence spectrum collection. Samples were excited at 500 nm, and we confirmed that this wavelength did not excite Cy5. All fluorescence spectra were collected in triplicate. Confirmed intensities were normalized by the Cy3 signal maximum at 566 nm after subtraction of baseline.

### Fluorescence polarization binding assays

Fluorescence polarization binding assays for ARPC2 rGQ were performed in 25 mm KCl, 0.25 m Arg/Glu, 20 mm Tris–HCl, pH 7.5, 0.1 mm EDTA, 0.1 mm TCEP, and 0.02% Tween 20. Ten nanomolar of 5′ fluorescein‐labeled RNA was mixed with proteins at concentrations ranging from 8000 to 0.488 nm, in black flat‐bottom 96‐well plates (Costar). Protein‐RNA mixtures (50 μL) were mixed at 100 RPM for 10 min, followed by incubation at 37 °C for 30 min, and then at 25 °C for 10 min. Fluorescence polarization was measured using a Cytation 5 with an excitation wavelength of 485 nm and an emission wavelength of 520 nm. We have shown that the total fluorescence intensities did not change during protein titration. Therefore, the dissociation constants (*K*_D_) were fitted using the quadratic equation with the assumption of one‐site interaction. The fluorescence polarization (*F*_p_) binding curves were fitted using the quadratic equation below, where the fitting parameters *F*_min_, *F*_max_, and *K*_D_ represent the fluorescence polarization baseline, plateau, and dissociation constant, respectively. [*P*_T_] represents the total protein concentration and [*L*_T_] represents the total RNA concentration (10 nm). Errors in dissociation constants were calculated based on three independent measurements.

(1) $$F_{p}=F_{\min}+\left(F_{\max}-F_{\min}\right)\frac{\left[\left(\left[P_{T}\right]+\left[L_{T}\right]+K_{D}\right)-{\left({\left(\left[P_{T}\right]+\left[L_{T}\right]+K_{D}\right)}^{2}-4\left[P_{T}\right]\left[L_{T}\right]\right)}^{0.5}\right]}{2\left[L_{T}\right]}$$

### Arg‐peptides synthesis and purification

(RQ)_10_, (RQ)_15_, (RQ)_20_, and R_30_ are products of GenScript. A Trp residue was attached to the C‐terminal end of the peptides for concentration determination using UV spectrometry.

(RGG)_13_, (RP)_15_, (RP)_20_, and (RE)_20_ peptides were synthesized using the automated flow peptide synthesis procedure described below. Fmoc‐protected amino acids (Fmoc‐Arg(Pbf)‐OH, Fmoc‐Glu(OtBu)‐OH, Fmoc‐Gly‐OH, Fmoc‐Ile‐OH, Fmoc‐Pro‐OH) were purchased from P3 Biosystems and dried under high‐vacuum prior to use. O‐(7‐azabenzotriazol‐1‐yl)‐N,N,N′,N′‐tetramethyluronium hexafluorophosphate (HATU, ≥ 97.0%) and (7‐azabenzotriazol‐1‐yloxy) tripyrrolidinophosphonium hexafluorophosphate (PyAOP, ≥ 97.0%) were purchased from P3 Biosystems and used directly. Acetonitrile (CH_3_CN), N,N‐dimethylformamide (DMF), and methylene chloride (CH_2_Cl_2_) were purchased from EMD Millipore. DMF was stored over AldraAmine trapping agents (for 1000–4000 mL DMF) prior to use. LC–MS grade CH_3_CN and water (H_2_O) were purchased from JT Baker. N,N‐diisopropylethylamine (DIPEA), piperidine, trifluoroacetic acid (TFA), formic acid (FA), and triisopropylsilane were purchased from Oakwood Chemicals. TentaGel® XV—Rink Amide (RAM) resin was purchased from Rapp Polymere.

Peptides were synthesized on an Automated Flow Peptide Synthesis (AFPS) instrument constructed in the Truex lab, based on the previously described system [24]. TentaGel® XV resin (150 mg, 0.26 mmol·g^−1^, 100–200 μm) with a rink amide (RAM) linker was suspended in ca. 5 mL of CH_2_Cl_2_ in a 5‐mL syringe (Torviq), pre‐equipped with a 7–12 μm membrane on top of the frit, and allowed to swell (15 min). The CH_2_Cl_2_ was drained and replenished with DMF (3 × 5 mL). The vessel was transferred to the solid‐phase peptide synthesis for fast‐flow peptide synthesis with the following conditions: Solutions were drawn from reagent bottles with three HPLC pumps to give a combined flow rate of 40 mL·min^−1^. Fmoc‐deprotection cycles were performed with two simultaneous streams: one 20 mL·min^−1^ stream of 40% piperidine and 2% formic acid and another 20 mL·min^−1^ stream of DMF. Coupling cycles were performed with three simultaneous streams: one 20 mL·min^−1^ stream of 0.38 m HATU (or 0.38 m PyAOP for Arg); a second 20 mL·min^−1^ stream of 0.4 m amino acid; and a third 2 mL·min^−1^ stream of DIPEA (neat). During amino acid coupling, all three pumps are simultaneously actuated for 11 mL (or 22 mL for Arg). Successive coupling and deprotection cycles are repeated for additional amino acids. Upon completion of the synthesis (ca. 2 h), the resin was rinsed with CH_2_Cl_2_ (5 × 5 mL) and aspirated until dry.

After the syntheses were complete, the peptides were cleaved from the resin under acidic conditions with a 3‐mL solution of trifluoroacetic acid (TFA), thioanisole, 1,2‐ethanedithiol, and triisopropyl silane (90/5/3/2). The crude peptides were isolated by trituration after the addition of chilled diethyl ether (ca. 45 mL, −80 °C), followed by mixing, centrifuging (5 min, 4 °C, 4000 rpm), and decanting the supernatant. The pellet was resuspended with 20% CH_3_CN in H_2_O, flash‐frozen in liquid nitrogen, and lyophilized. The lyophilized powder was analyzed by analytical LC–MS on an Agilent LC‐QTOF (6545XT) system equipped with Poroshell 120 EC‐C18 (2.7 μm, 2.1 × 100 mm) at 40 °C, using a 1–91% linear gradient over 10 min of CH_3_CN in H_2_O with 0.1% FA at 0.5 mL·min^−1^. Purification was achieved by preparative RP‐HPLC on an Agilent Zorbax SB‐C18 Prep HT column (7 μm, 21.2 × 50 mm) at 60 °C, using a 0–30% linear gradient over 30 min of CH_3_CN in H_2_O with 0.1% TFA at 15 mL·min^−1^. Lyophilization of the purified fractions gave the peptides as a white powder in 1–2% yield (1–7 mg) based on a 40 μmol synthesis scale. The synthesized peptides were further dialyzed into water to remove residual shorter species and contaminants. The identity and purity of these peptides were confirmed by mass spectroscopy.

### Molecular dynamics (MD) simulation and analysis

Molecular dynamics simulations were performed using Amber with the ff19SB force field for proteins and OL3 parameters for RNA, combined with the OPC water model [25, 26, 27]. The system was solvated in an octahedral OPC water box (10 Å) and neutralized with Na^+^ ions. It underwent restrained energy minimization for 5000 steps, heating from 0 to 300 K over 50 ps in the NVT ensemble, followed by 50 ps equilibration in the NPT ensemble at 1 atm with reduced positional restraints. Production MD simulations were run for over 500 ns under periodic boundary conditions in the NPT ensemble, using a 2‐fs timestep and Langevin thermostat. Coordinates were saved every 150 ps for analysis. Backbone RMSD was calculated to confirm system equilibration. Hydrogen bonds between protein and RNA were analyzed using donor‐acceptor distance and angle cutoffs of 3.5 Å and 120°, respectively. Salt bridges and stacking interactions were evaluated based on distances below 4 Å and angular deviations within 30°. Simulations were repeated three times using different starting conformations.

### Single‐molecule Förster resonance energy transfer (smFRET)

The fluorescently labeled hairpin DNA was purchased from IDT. The target RNA oligonucleotide was purchased from Dharmacon and deprotected following the manufacturer's protocol. DNA and RNA oligos were desalted with Bio‐Spin P‐30 gel columns (BioRad, catalog# 7326232) and stored in nuclease‐free water before annealing. RNase H and 10X RNase H reaction buffer were purchased from NEB (catalog# M0297S). The DNA–RNA hybrid duplex was annealed by mixing 10 μm of the DNA and 10.5 μm of RNA in 10 mm Tris–HCl, 100 mm NaCl at pH 7.5, incubated at 95 °C for 3 min, then immediately cooled to 4 °C. The smFRET experiments were performed with a prism‐type Total Internal Reflection Fluorescence microscope at room temperature (25 ± 2 °C). All instrumentation, slide preparation, and general smFRET experiment procedures were conducted as reported earlier [28, 29]. The oxygen scavenging imaging buffer is composed of 10 mm Tris, 100 mm NaCl, 0.5% v/v D‐glucose, 1 mg·mL^−1^ glucose oxidase, 10 mm Trolox, and 2 μg·mL^−1^ catalase. Data were acquired at a 100 ms per frame rate and analyzed as reported earlier. Real‐time RNase H cleavage reactions were performed by introducing 100 μL of imaging buffer containing 0.1 unit·μL^−1^ RNase H and 1× RNase H reaction buffer. Real‐time SRSF1 RS binding assay was performed by introducing 100 μL of imaging buffer containing 20 μm of SRSF1 RS. Each FRET histogram was generated from 15 to 20 individual movies for 2 s long, which contained a total of 6000 or more individual molecules of a single Cy3‐Cy5 pair. The reported time points post protein introduction were the beginning time of recording the set of images. Image collection for each image set used in one histogram usually takes 3 ± 2 min. Three or more experimental repeats were performed for each experiment.

## Results and Discussion

### Unfolding of GQ by SR and SR‐related proteins

The stability of rGQs mainly depends on the layers of G‐tetrads [4]. We selected a model rGQ structure that is representative of G‐tetrad motifs to test the unfolding activity of SR or SR‐related proteins. Among the mRNA transcripts regulated by SRSF1, we analyzed 1576 mRNA transcripts for the occurrence of rGQs with 2‐, 3‐, or 4‐layer G‐tetrads [30]. Of these transcripts, 1212 sequences contain 2‐layer rGQs, 184 sequences contain 3‐layer rGQs, and only 8 sequences contain 4‐layer rGQs (Fig. 1B). As 2‐layer rGQs are significantly less stable than 3‐layer rGQs and 4‐layer rGQs occur rarely, we selected the 3‐layer rGQ in the 5′ untranslated region of ARPC2 as a model to test the unfolding activity.

To monitor the unfolding of ARPC2 rGQ, we labeled the rGQ with Cy5 and Cy3 at the 5′ and 3′ ends, respectively. rGQ unfolding will reduce the resonance energy transfer efficiency and retain the fluorescence energy in Cy3 (maximum around 566 nm), resulting in decreased peak intensity for Cy5 (maximum around 672 nm) after normalization of the fluorescence spectrum at the Cy3 maximum. Our previous research has verified this method and confirmed that the change of relative Cy5/Cy3 signal is due to the FRET efficiency instead of fluorescence quenching [12].

We have previously shown that SRSF1 can unfold rGQs. To test whether other SR proteins can also unfold rGQs, we selected SRSF9 and SRSF3. SRSF9 has the shortest RS domain and SRSF3 has an RS domain that is just longer than SRSF1 (Fig. 1C). If these three SR proteins can unfold ARPC2 rGQ, we predict that other SR proteins also have unfolding activity because they have longer RS regions. We found that SRSF9 also reduced the Cy5 signal, although the magnitude is smaller than that of SRSF3, suggesting that these two SR proteins can also unwind ARPC2 rGQ (Fig. 1D).

To further test whether SR‐related proteins have this activity, we chose BAD1 and BAD2 of U1‐70K, as well as U2AF1 and U2AF2 (Fig. 1D). We found that BAD1 of U1‐70K and U2AF1 unfolded ARPC2 rGQ, while BAD2 or U2AF2 had no such activity. From these SR or SR‐related proteins, we found that the length of RS and the number of Arg were important for the unfolding activity. For example, SRSF3, BAD1, and U2AF1 have stronger unfolding activities than SRSF9. In addition, acidic residues attenuated the activity, as shown in the case of BAD2, which has a balance of acidic and basic residues.

### Unfolding of GQ by Arg‐rich peptides

**Fig. 2:**
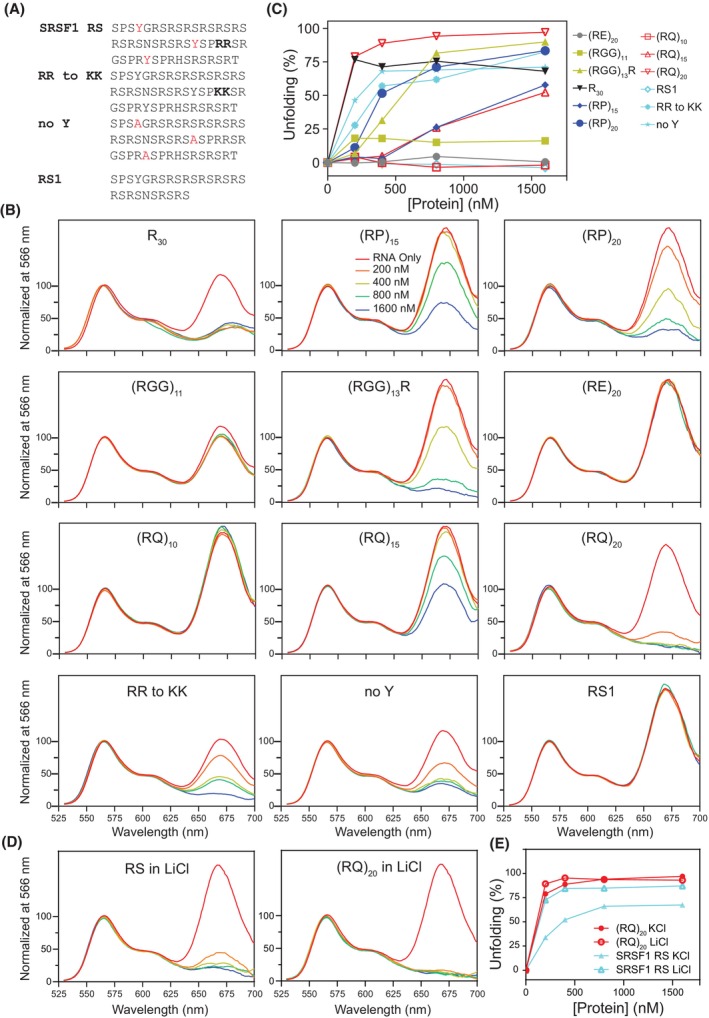
Impact of amino acid composition and peptide length on the rGQ‐unfolding activity of Arg‐rich peptides. (A) Sequences of SRSF1 RS and various mutants with mutated sites highlighted in red. (B) Fluorescence spectra of Cy5/Cy3‐labeled ARPC2 rGQ unfolding in 25 mm KCl normalized to the Cy3 emission maximum at 566 nm. All spectra were collected with excitation at 500 nm and represent the average of five scans. ARPC2 rGQ was used at a concentration of 200 nm, and the concentrations of Arg‐rich peptides are indicated. (C) ARPC2 rGQ unfolding curves plotted with data from panel B. (D) ARPC2 rGQ unfolding in 25 mm LiCl with all other settings the same as panel B. (E) ARPC2 rGQ unfolding curves plotted with data from panel D. The unfolding percentage in panels C and E at a given concentration X was calculated as (F_670 nm, 0 nm protein_ − F_670 nm, X nm protein_)/F_670 nm, 0 nm protein_.

Our previous experiment showed that the peptide containing RA motifs also unfolded ARPC2 rGQ, suggesting that the unfolding activity can be extrapolated to other Arg‐rich peptides [12]. To investigate the impact of amino acid sequence composition and length of Arg‐rich peptide on the unfolding activity, we evaluated peptides containing amino acid repeats of RR, RE, RQ, and RGG (Fig. 2A). These sequences provide contexts of Arg adjacent to basic, acidic, polar, and hydrophobic residues, respectively. We also tested a peptide containing amino acid repeats of RP, providing a context of Arg amino acids with restricted backbone flexibility. We also investigated the impact of amino acid length on the unfolding activity by comparing peptides of varying length. These peptides represent the sequence contexts for most Arg‐rich peptides in the human proteome, particularly for the RGG motif that is common in RNA‐binding proteins, including FUS, hnRNP A1, hnRNP U, and TAF15.

Except for the RE peptide, we found that most Arg‐rich peptides could unfold the ARPC2 rGQ motif in a dose‐dependent fashion and that pronounced activity was observed with the longer peptides (Fig. 2B). Comparison of (RP)_20_ and (RQ)_20_ suggests that (RP)_20_ is less active as the Cy5 signal decreases more slowly than that of (RQ)_20_ (Fig. 2C). We further selected the RQ peptides to test the impact of length on the unfolding activity, finding that 10 repeats of RQ were unable to open rGQ, but 15 repeats of RQ were sufficient. These results are consistent with RGG peptides, which also require at least 13 repeats to unfold ARPC2 rGQ. The peptide with RE repeats is unable to open rGQ, possibly because acidic residues neutralize the positive charge of Arg residues and reduce binding to RNA.

We also mutated SRSF1 RS to confirm our findings in the model peptides. We mutated two consecutive Arg to Lys and found that these mutations had a mild impact. We also mutated all three Tyr residues to Ala (no Y in Fig. 2B,C) and found that these mutations had a milder impact than the RR to KK mutant (Fig. 2B,C). Consistent with our findings that at least 13 Arg residues are needed, the truncated SRSF1 RS (RS1), which has 10 Arg residues, has no rGQ‐unfolding activity. rGQ is less stable in Li^+^ buffer. Accordingly, RS and (RQ)_20_ showed greater rGQ‐unfolding activity in Li^+^ than in K^+^ (Fig. 2D,E).

### The unfolding activity of RS is specific to rGQ

**Fig. 3:**
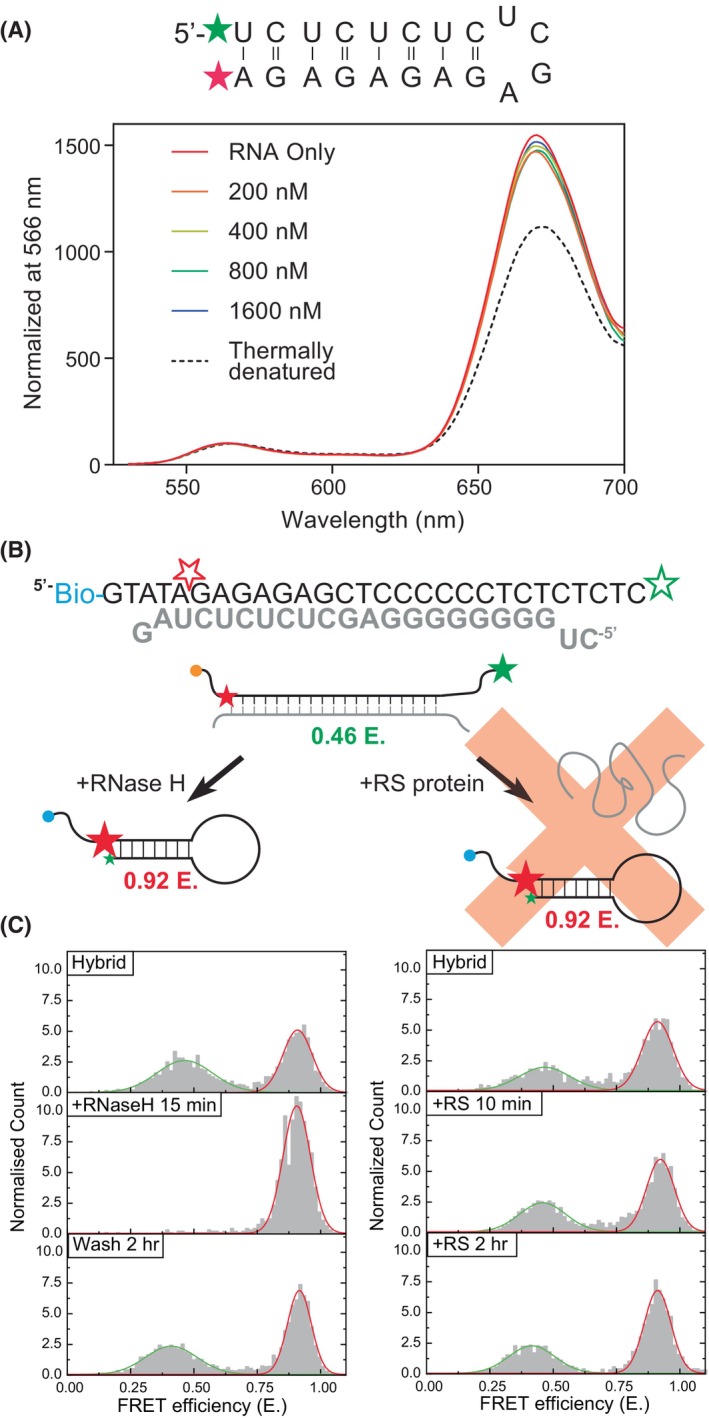
The RS unfolding activity is specific to rGQ. (A) SRSF1 RS cannot unfold stem‐loop RNA. The green and red stars indicate labeling sites for Cy3 and Cy5, respectively. The spectra were the average of five scans. (B) smFRET assay for SRSF1 RS binding with G‐rich RNA in double helix. Experimental design and corresponding FRET efficiency of each conformation. (C) smFRET histogram of the DNA–RNA hybrid before protein addition (top panels), after RNase H cleavage, buffer wash, and 20 μm SRSF1 RS domain incubation. smFRET experiments were performed in three replicates.

To test whether the unfolding activity also applies to other structured RNA, we tested stem‐loop forming RNA and DNA/RNA hybrid. As shown in Fig. 3A, RS has no unfolding activity to stem‐loop RNA (Fig. 3A).

We designed a molecular beacon system to test whether SRSF1 RS can unfold to G‐rich RNA in a long double‐helix. A 24‐nt RNA oligonucleotide (rCUG_8_AGCUCUCUCUCUAG) is annealed to a complementary DNA strand (/biotin/GTATA/Cy5/GAGAGAGCTC_6_TCTCTCTC/Cy3/) to form a DNA–RNA hybrid duplex with an 18‐bp standard double‐helix structure and a GT wobble base pair (Fig. 3B), which separates the dye pair on the DNA to the two ends of the double‐helix and gives low FRET efficiency (E) of 0.46 ± 0.02. As shown in the smFRET histograms (Fig. 3C top panels), the annealed DNA–RNA mixture has 39 ± 10% of the population in the 0.46 E. duplex form, while the rest of the signal shows a high E. of 0.92 ± 0.01 for the DNA hairpin. When 0.1 unit·μL^−1^ of RNase H was introduced to the annealed mixture, all the 0.46 E. signal (hybrid duplex) moved to 0.92 E. (DNA hairpin) within 10 min (Fig. 3C left middle panel). Adding 20 μm of SRSF1 RS or equal volume of buffer wash only caused less than 5% decrease of the 0.46 E. population after 2 h of incubation (Fig. 3C), which indicated that SRSF1 RS cannot remove the RNA from hybrid duplex. Analysis of long smFRET traces showed no significant change of dynamic behavior of the FRET signal or protein induced fluorescent enhancement, which indicated that SRSF1 RS cannot remove RNA from the hybrid duplex. It is noteworthy that the percentage of DNA–RNA hybrid duplex at the beginning of each experiment varied with the time between complex annealing and surface immobilization for smFRET experiments. The percentage decline in response to the RNase H or SRSF1 RS was independent of the initial ratio of the hybrid duplex. Therefore, the reported binding above was specific to G‐rich RNA in a GQ conformation.

### Interactions important for rGQ unfolding by Arg‐rich peptides

**Fig. 4:**
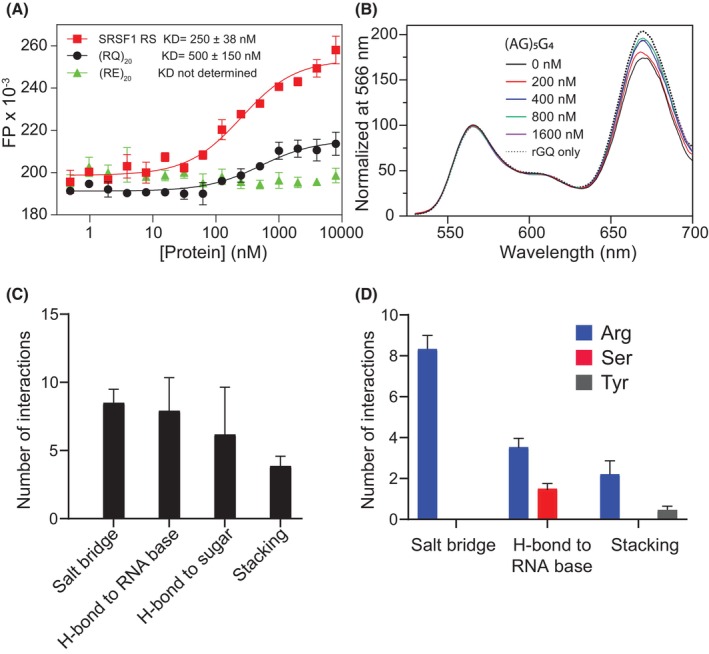
Fluorescence polarization binding assays and molecular dynamics analysis of SRSF1 RS interactions with unfolded ARPC2 rGQ. (A) Fluorescence polarization binding assays for ARPC2 rGQ and Arg‐rich peptides. ARPC2 rGQ (10 nm) was labeled at 5′ with fluorescein. Error was estimated from three replicates. (B) Single‐stranded RNA (AG)_5_G_4_ competes with rGQ for SRSF1 RS binding and therefore inhibits the unfolding activity of RS. The fluorescence spectrum of rGQ alone is shown as a reference. (AG)_5_G_4_ of varying concentrations (0–1600 nm) was titrated into the mixture of 200 nm of RS and 200 nm of Cy5/Cy3‐labeled ARPC2 rGQ. The spectra were an average of five scans. (C) Number of interactions per frame between the RS domain of SRSF1 and the unfolded rGQ, calculated over a 150‐ns postequilibration trajectory. (D) Contributions of Arg, Ser, and Tyr residues to salt bridges, hydrogen bonds, and stacking interactions with rGQ. Only hydrogen bonds formed between RS residues and RNA bases are shown. Error bars represent the standard deviation of three replicate MD simulations.

We showed that the unfolding activity is driven by Arg residues and inhibited by acidic amino acids. We reasoned that acidic amino acids neutralized the positive charge of Arg and therefore abolished RNA binding. To test this, we measured binding affinity of rGQ with SRSF1 RS, (RQ)_20_, and (RE)_20_ (Fig. 4A). Consistent with the unfolding results, SRSF1 RS and (RQ)_20_ bound with rGQ, whereas (RE)_20_ did not. We also performed competition assays using a single‐stranded RNA (AG)_5_G_4_ that does not form rGQ but binds with SRSF1 RS. As shown in Fig. 4B, (AG)_5_G_4_ reduces the unfolding activity of SRSF1 RS. All these results suggest that Arg‐rich peptides unfold rGQ structures primarily through direct RNA interactions.

The free energy of peptide–RNA interactions must be sufficient to overcome the intrinsic stability of rGQs, although quantifying and comparing these energies remains challenging. Molecular dynamics (MD) simulations provide a practical way to examine these interactions. We performed MD simulations of the SRSF1 RS domain with ARPC2 rGQ, using extended conformations of both partners, as suggested by our FRET unfolding assays, which showed minimal Cy5 signal. Over more than 500 ns of simulation (Fig. 4), we observed abundant salt bridges, frequent hydrogen bonds between Arg side chains and guanine bases, and occasional base‐stacking with Arg or aromatic residues. Salt bridges predominated, followed by hydrogen bonds to RNA bases and the ribose backbone (Fig. 4C). Among the three most abundant residues in SRSF1 RS—Arg, Ser, and Tyr—Arg contributed most to RNA binding, forming substantially more base‐specific hydrogen bonds despite Ser being more numerous (Fig. 4D). This supports our experimental conclusion that Arg residues are the primary drivers of rGQ unfolding. For a three‐layer rGQ, 12 guanosines form a Hoogsteen base pairing network, requiring sufficient Arg residues to interact with these guanosines and maintain the RNA in an unfolded state. Notably, Ser residues also contributed by forming hydrogen bonds with RNA, consistent with our observation that RQ peptides are more potent rGQ unfoldases than RGG or RP peptides.

## Conclusions

In this study, we show that rGQ‐unfolding activity is a common feature of many SR proteins, SR‐related proteins, and other proteins with Arg‐rich regions. These proteins likely represent important—and previously underappreciated—contributors to the global regulation of rGQ structures in cells, with potential impacts on RNA splicing and translation that warrant further investigation. Arginine residues are the principal drivers of the unfolding activity, and acidic amino acids attenuate this activity by neutralizing Arg's positive charges. The total number of Arg residues, that is, the length of the Arg‐rich segment, is a key determinant: For ARPC2 rGQ, at least 13 Arg residues are required for unfolding. We further show that Arg‐rich regions unfold rGQ but not stem‐loop RNA or RNA/DNA hybrids. Arg residues promote unfolding through salt bridges and hydrogen bonds with nucleotide bases and ribose, as well as cation–π stacking. These findings not only expand our understanding of rGQ regulation but also provide a new, helicase‐independent approach to manipulate rGQ folding, offering a straightforward strategy to control the structural status of rGQ‐forming RNA sequences.

## Author contributions

NIUDS, PK, H‐TL, and JZ designed the study, analyzed the data, and drafted the paper. NIUDS, PK, JZ, MIRR, JKK, ZZ, TP, and NL performed the experiments. CC wrote scripts and analyzed the data. H‐TL and NT validated the data and acquired funding. JZ validated the data, acquired funding, and administrated the project.

## Data Availability

All data related to this manuscript are available from the corresponding authors.
